# Right Upper Lobe Shadow and Tracheobronchopathia Osteochondroplastica Confined to Right Main Bronchus: A Case Report and Literature Review

**DOI:** 10.1155/2015/368485

**Published:** 2015-11-22

**Authors:** Stylianos A. Michaelides, George D. Bablekos, George Ionas, Stephanie Vgenopoulou, Maria Chorti

**Affiliations:** ^1^Department of Occupational Lung Diseases and Tuberculosis, “Sismanogleio” General Hospital, Sismanogleiou 1 Street, Maroussi, 15126 Athens, Greece; ^2^Faculty of Health and Caring Professions, Technological Educational Institute (TEI) of Athens, Agiou Spyridonos Street, Egaleo, 12243 Athens, Greece; ^3^1st Department of Thoracic Medicine, “Sismanogleio” General Hospital, Sismanogleiou 1 Street, Maroussi, 15126 Athens, Greece; ^4^Department of Pathology, “Sismanogleio” General Hospital, Sismanogleiou 1 Street, Maroussi, 15126 Athens, Greece

## Abstract

Tracheobronchopathia osteochondroplastica (TO) is a well documented benign entity of endoscopic interest. We describe a case of 76-year-old patient who presented with fever, cough, purulent sputum during the past four days, and presence of an ovoid shadow in right upper zone of his chest X-ray. Medical history included diagnosis of colon diverticuli identified by colonoscopy 3 months ago. Chest CT revealed a compact elongated lesion containing air-bronchogram stripes. Bronchoscopy showed normal upper airways and trachea but presence of unequal sized mucosal nodules, protruding into the lumen, along the entire length of the right main bronchial mucosa. No other abnormal findings were detected. Moreover, brushing and washing smears from the apical segment of right upper lobe (RUL), where the compact lesion was located, were negative for malignancy. Biopsy from the mucosal nodules of right main bronchus showed presence of cartilaginous tissue in continuity through thin pedicles with submucosal cartilage. This finding posed the diagnosis of TO while RUL lesion was cleared by antibiotic treatment. Case is reported because, to our knowledge, it represents a unique anatomic location of TO which was confined exclusively in the right main bronchus mucosa without affecting trachea.

## 1. Introduction

Tracheobronchopathia osteochondroplastica (TO) is an uncommon disorder that was first described in the middle of the 19th century and has a higher incidence in northern Europe [1]. It is an idiopathic, nonmalignant condition characterized by the presence of submucosal cartilaginous or osseous nodules overlying the cartilaginous rings of the large airways [2]. Clinical presentation is variable, ranging from complete lack of symptoms to cough, hemoptysis, breathlessness, or recurrent chest infections [3]. It can more rarely cause tracheal stenosis resulting in difficult intubation of the patient [4–6]. Few isolated cases of a single solitary nodule amenable to cartilaginous protrusion of the same pathology as in TO have been reported in lobar [7] or subsegmental [8] bronchi, being causes of atelectasis or pneumonia. Cases of TO are extremely rare in childhood [9] or may have a familial incidence [10]. The vast majority of cases of TO are only diagnosed at autopsy having been asymptomatic during life [10].

We report the case of a male patient, who was investigated for a compact lesion in his right upper lobe (RUL) zone and eventually diagnosed to have lesions of TO only in his right main bronchus. The particularity about this case is the presence of these nodules exclusively along the whole length of the right main bronchial mucosa, without involvement of the trachea. Also, to the best of our knowledge, there have been only two cases in literature where a solitary nodule of TO was reported: one was confined to the RUL bronchus [7] and the other to the right subsegmental (B3b) bronchus [8]. We further attempt to enrich the relevant literature by focusing on the diversity of anatomic locations of TO.

## 2. Case Presentation

**Figure 1:**
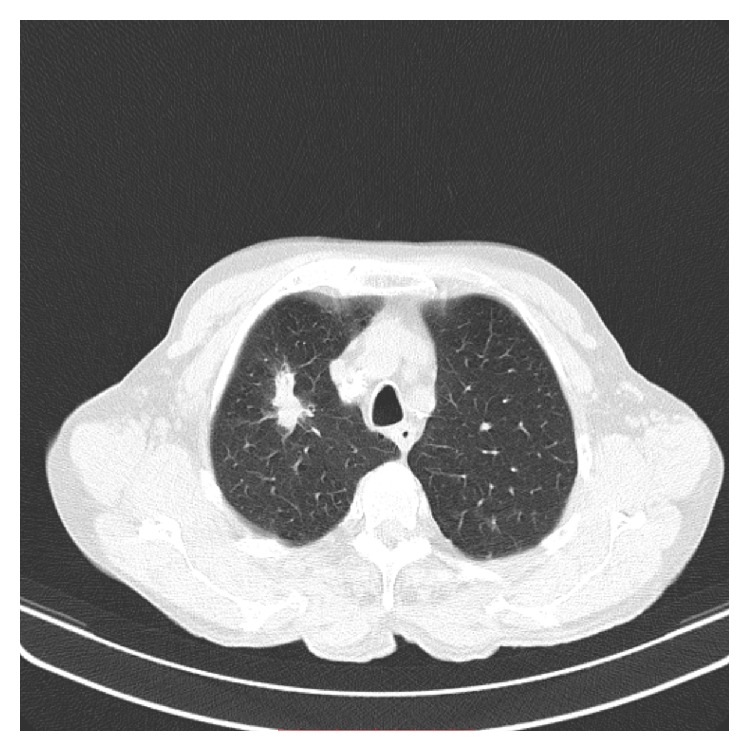
CT-scan of the chest showing an irregular elongated shadow in apical segment of right upper lobe.

**Figure 2:**
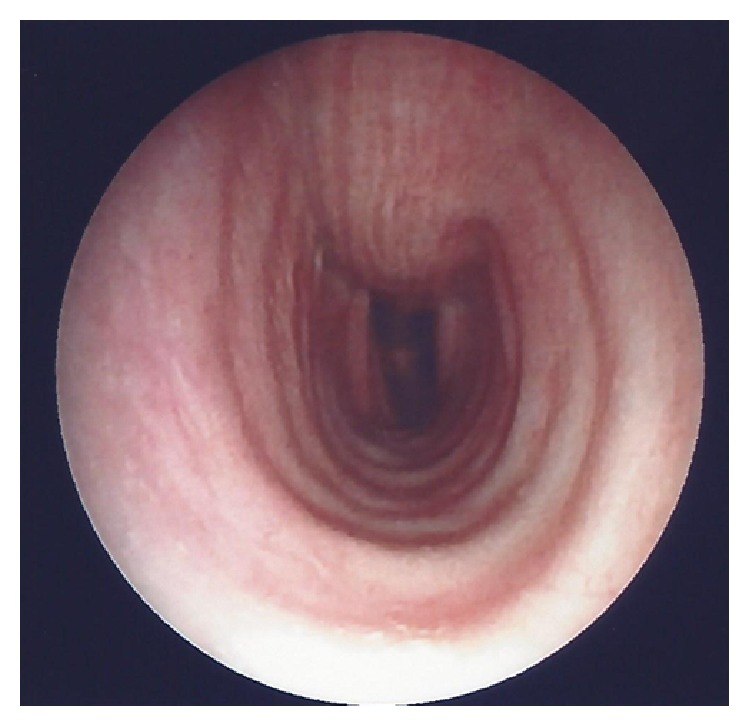
Bronchoscopic image of the patient's normal tracheal mucosa.

**Figure 3:**
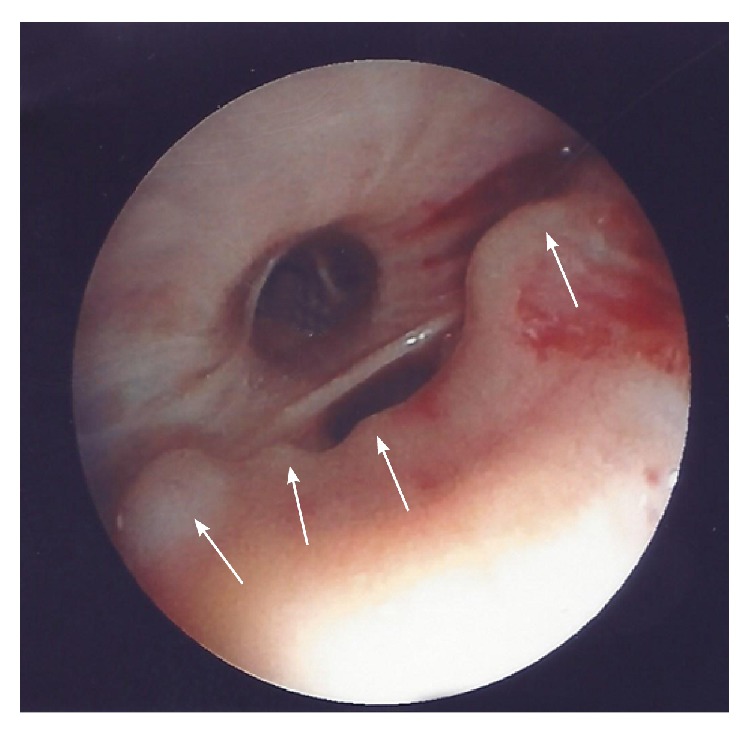
Bronchoscopic image of the patient's right main bronchus showing unequal nodular protrusions of the mucosa.

**Figure 4:**
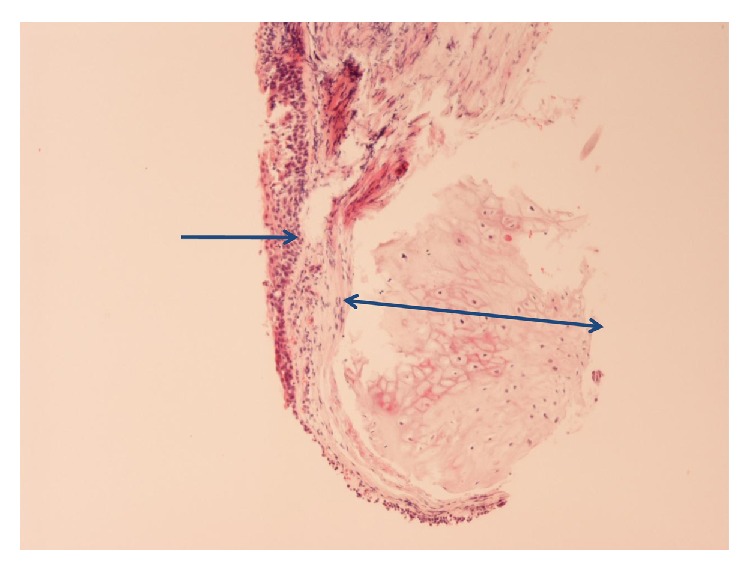
Histopathologic image of patient's bronchial wall showing presence of cartilaginous tissue in submucosa, compatible with tracheobronchopathia osteochondroplastica (Hematoxylin-Eosin stain ×40).

This is about a 76-year-old heavy smoking male patient who presented with a history of 4-day duration of fever, cough, and purulent sputum. He was admitted to hospital because of presence of an irregular rather compact elongated shadow in the right upper zone of his chest X-ray. The patient had been a farmer for all previous years. His past medical history included an operation for inguinal hernia 15 years ago. Three months ago, he underwent colonoscopy for abdominal discomfort and investigation of chronic microcytic anemia which proved to be due to diverticuli in descending colon and sigmoid. An abdominal Computed Tomography (CT-scan) further revealed small cortical cysts on his left kidney as well as a tiny calculus in the middle calyx of the same kidney. All full blood and biochemical tests were within normal limits except for mild hypochromic anemia (Htc = 38%), a moderate neutrophilic leucocytosis, and elevation of C-reactive protein (CRP = 18.9 mg/L). A chest CT-scan showed an elongated irregular shadow with traces of air-bronchogram stripes in it (Figure 1) for which the patient underwent fiberoptic bronchoscopy (FOB) to exclude malignancy. Upper airways and trachea were normal (Figure 2) but right main bronchus was roughened by multiple unequal sized nodules with no signs of infiltrated mucosa along the bronchus' entire length (Figure 3). No other abnormal findings were detected in the bronchial tree down to the level of subsegmental branches. Washings and brushings were obtained from the apical segment of right upper lobe (RUL) where the compact lesion was located. Several biopsies were also taken from the right main bronchus mucosal nodules. The patient was administered (iv) Cefuroxime Sodium, 750 mg tid for 5 days, and continued with per os Cefuroxime axetil 500 mg bid for another 5 days with almost radiological clearance of the RUL shadow. Biopsy from nodules of right main bronchus was sent for histopathologic diagnosis. Microscopy revealed that the nodules consisted of cartilage situated between the normal cartilage and the surface epithelium of the bronchus. Step sections showed that there was indeed continuity through narrow pedicles, in concordance with the view that the condition represented multiple ecchondroses of the bronchial cartilages (Figure 4). These findings posed the diagnosis of TO while RUL lesion proved to be pneumonia.

## 3. Discussion

Our patient had been a lifelong heavy smoker presenting with a rather compact irregular elongated shadow in his right upper lobe. It was therefore needed to exclude presence of lung cancer. This is why he promptly underwent fiberoptic bronchoscopy (FOB) to exclude this possibility. Presence of nodules along the entire length of his right main bronchus but complete absence of these lesions anywhere else in his bronchial tree warranted histological identification of these lesions. Obtaining washings and brushings from the apical segment of RUL where the irregular compact lesion appeared in his chest CT was also needed. Chest CT can sometimes provide evidence of presence of TO by displaying calcification of the tracheal and bronchial wall with irregular inner lining [2]. In our patient, the crucial CT finding was a compact RUL shadow. His response to antibiotics and clearance of the RUL lesion proved the ovoid shadow to be pneumonia. Although pneumonia associated with TO has been reported due to relative stenosis of a bronchus [8], this does not seem to be the case in our patient for the following reasons: (a) despite the fact that TO nodules were protruding along the entire length of the right main bronchus, they did not significantly obstruct its lumen to cause retention of secretions and (b) it is well known that clearance of secretions in upper lobes is aided by gravity [11]. However, impaired mucociliary clearance has been thought to exist, at least locally, in cases with TO [12]. This is in accordance with another study [11] reporting lack of cilia continuity in damaged trachea segments due to TO, thus contributing to retention of respiratory secretions. Moreover, TO was reported for the first time in literature in 1857 by Wilks [13]. Two other studies [14, 15] showed that cases registered from 1857 to 1974 [14] and to 1998 [15] were 254 [14] and 370 [15], respectively.

Also, to the best of our knowledge, by reviewing the relevant literature from 1987 to present, 62 cases of TO occurring in different anatomic positions were published [2–8, 11, 16–20]. Specifically, TO was found to develop: (1) 22 cases in trachea [3–5, 16, 18–20], (2) 2 cases where nodules were located from the first tracheal ring to the proximal main stem bronchi [17], (3) 22 cases in trachea and both main stem bronchi [2, 6, 11, 16, 19, 20], (4) 8 cases in trachea and main stem bronchus [3], without determining the number of the right and/or the left main stem bronchi, (5) 2 cases in trachea and left main stem bronchus [2, 20], (6) 1 case in trachea and left lingular bronchus [20], (7) 2 cases in trachea and right main stem bronchus [20], (8) 1 case in trachea and right intermedius bronchus [20], (9) 1 case in RUL bronchus [7], and (10) 1 case in right subsegmental B3b bronchus [8].

The exact cause [1, 19], molecular basis [16], and pathogenesis [19] of TO are all unknown but it has been hypothesized that TO might be related with several chronic inflammatory conditions such as infections, trauma, silicosis, or amyloidosis [11]. A case of TO associated with allergic bronchopulmonary aspergillosis which is also a chronic inflammatory condition has been recently reported [2]. According to some other studies, TO was further supposed to be related to bronchial asthma [21, 22] and ozaena [22, 23]. In TO, the posterior membranous wall of the trachea is always spared since this condition has its origin in the cartilage and this is a differentiating characteristic of TO from other granulomatous conditions or amyloidosis [7, 8]. Coexistence of TO with atrophic rhinitis and botryomycosis has also been reported in literature [18].

Theories regarding formation of TO do exist and include, on the one hand, the hypothesis that the initial lesions are chondromas which can calcify and ossify thus producing nodules [2], and, on the other hand, the theory of metaplasia according to which the nodules originate from the ossification of elastic tissue [2]. Bone morphogenetic protein-2 (BMP-2) and transforming growth factor-beta 1 (TGF-beta 1) are thought to be potent inducers for new bone formation. This has been supported by the fact that positive BMP-2 immunoreactivity was detected in numerous mesenchymal cells and chondroblasts lining the nodules in the tracheal submucosa [17].

The incidence of TO was reported to be 1 in 2000 [10] and 5 in 3500 [24] bronchoscopies. In another study [25], the incidence of TO was estimated to be 2 to 7 in 1000 cases. Characteristic endoscopic findings supporting TO diagnosis include hard whitish spicules, deriving from both the anterior and lateral tracheal wall, which project into the tracheal lumen [4], while main bronchi and larynx are less often affected [13]. Besides, mean ages for men and women concerning TO emergence were 42 and 51 years, respectively [26].

Differential diagnosis of TO is made from Wegener granulomatosis [2], sarcoidosis [4], tracheal and mediastinal tumors [11], tracheal amyloidosis [11], respiratory papillomatosis [11], and relapsing polychondritis [11]. Clinical symptoms of the disease are chest pain [19], cough [16, 19], dyspnea [16, 19], hemoptysis [16, 19], hoarseness [19], wheeze [16], and recurrent infections [19], with the main symptoms being hemoptysis [27] and chronic cough [28].

Conservative treatment of TO includes maintenance of airway humidity and mucolytics as well as administration of antibiotics for respiratory infections [2, 11]. Moreover, although laser ablation, rigid bronchoscopy, and stent placement can be additionally used to improve clinical condition of symptomatic patients [3], surgery seems to be the treatment of choice for TO [4]. This is enhanced by another study supporting that 19% of patients with TO presented simultaneously malignant tumors [29], while laser intervention does not contribute to complete effacement of the disease [11]. Surgery is further indicated if tracheal lumen is severely obstructed from TO nodules [19, 30]. In addition, surgery constitutes the method of choice to treat TO, when disease is associated with bleeding [19] or recurrent infection [19]. In our case, surgery was not a therapeutic option since TO nodules were confined to right main bronchus without obstructing the orifice of RUL so as to be considered responsible for occurrence of pneumonia.

Particular attention should be paid by anesthesiologists as difficult intubation can occur [6] in the presence of TO. For patient's safety, the endotracheal tube should be of appropriate size taking into account airway dimensions by means of preoperative imaging and/or bronchoscopy [6]. Within the context of medical treatment for TO, radiologists, pulmonologists, and general physicians should be experienced with imaging techniques such as Computed Tomography (CT) and findings of bronchoscopy, for making prompt diagnosis and treatment in order to differentiate TO from other entities [2]. Such entities could be lung cancer, tuberculosis, or sarcoidosis, the exclusion of which is of paramount importance for patient's clinical outcome, given that hemoptysis and chronic cough usually accompany the above diseases.

Diagnosis of TO can be made from chest CT-scan when small ovoid radioopaque protrusions are detected in the lumen of the trachea. Bronchoscopy is the main modality of diagnosing TO since the nodules characteristically have a smooth shape and emerge from tracheal rings but never involve the membranous part. Biopsy of the nodules definitely poses histopathologic diagnosis of this entity. Particularly, nodules composed of focal calcifications covered with normal mucosa and/or heterotopic bony and cartilaginous structures can be histopathologically observed [31]. Diagnosis based only on clinical presentation and chest X-ray is rarely possible.

## 4. Conclusion

Since TO is expected to involve tracheal mucosa, the anatomic location of similar nodules in only one main bronchus should not mislead diagnosis. This is because similar endoscopic appearance may be mimicked by several pulmonary infectious or neoplastic diseases.
